# Accommodating Celiac Disease in Higher Education: Evidence-Informed National Recommendations

**DOI:** 10.3390/nu18020294

**Published:** 2026-01-16

**Authors:** Vanessa Weisbrod, Meghan Donnelly McKeon, Emma Kowzun, Marilyn Grunzweig Geller, Jackie Jossen, Marisa Gallant Stahl, Maureen M. Leonard, Mary Shull, Janis Arnold, Jennifer Kumin, Sharon Weston, Anne R. Lee, Mary Vargas, Dale Lee, Allyson West, Catherine Raber, Katherine Vera Sachs, Ritu Verma

**Affiliations:** 1 Celiac Disease Foundation, Woodland Hills, CA 91364, USA; meghan.donnelly@celiac.org (M.D.M.);; 2 Celiac Disease Center, Columbia University Medical Center, New York, NY 10032, USA; 3 Children’s Hospital Colorado, Aurora, CO 80045, USA; 4 Mass General Brigham for Children, Boston, MA 02114, USA; 5 Boston Children’s Hospital, Boston, MA 02115, USA; 6 Wentworth Douglass Hospital, Dover, NH 03820, USA; 7 Stein & Vargas, LLP, Frederick, MD 21704, USA; 8 Seattle Children’s Hospital, Seattle, WA 98105, USA; 9 Harvest Table Culinary Group, Philadelphia, PA 19103, USA; 10 Children’s National Hospital, Washington, DC 20010, USA; 11 Division of Hospital Medicine, School of Medicine, University of Colorado, Denver, CO 80203, USA; 12 Celiac Disease Center, University of Chicago, Chicago, IL 60637, USA

**Keywords:** celiac disease, gluten-free diet, students, universities, chronic disease, college, autoimmune, food service, higher education

## Abstract

**Objectives:** We aimed to develop expert-informed recommendations for colleges and universities to support students with celiac disease (CeD) managing a gluten-free (GF) diet. **Methods:** A multidisciplinary panel of 40 stakeholders, including physicians, dietitians, a disability rights attorney, university staff, and students, was convened by the Celiac Disease Foundation to create expert-based and experience-informed recommendations. Over a 6-month period, the group conducted literature reviews, stakeholder interviews, and expert consensus discussions to identify common barriers and accommodations aligned with federal disability law. The expert panel collaboratively developed and revised an initial set of recommendations. Two rounds of structured voting were held during which panelists provided feedback to refine content and ensure clarity. All final recommendations were adopted with at least 90% of panelists voting in support. **Results:** The panel identified 24 accommodations across four domains: academics, housing, dining, and campus life. Academic recommendations include flexibility for illness-related absences, support for remote learning, and classroom modifications. Housing recommendations emphasize access to priority placement, appropriate appliances, and proximity to safe dining. Dining accommodations address GF food availability, ingredient transparency, staff training, and meal plan flexibility. Campus life recommendations ensure full participation in athletics, study abroad, social events, and internships, with supports for psychosocial well-being. **Conclusions:** This manuscript presents the first expert-informed recommendations focused specifically on the needs of college students with CeD. These recommendations are intended to support institutions as they develop strategies to enhance access to GF food, quality of life, educational supports, and student experience for those living with this chronic autoimmune condition.

## 1. Introduction

Celiac disease (CeD) is a chronic genetic autoimmune condition that affects at least 1% of the global population [1]. In patients with CeD, ingesting even small amounts of gluten triggers an immune response that damages the small intestine, leading to malabsorption, nutrient deficiencies, and a range of physical and cognitive symptoms. The only treatment is lifelong adherence to a strict gluten-free (GF) diet [2].

For college students, managing CeD presents unique challenges. Dining halls, shared kitchens, and unpredictable food environments create risk of unintentional gluten exposure. Social stigma, inconsistent accommodations, and a lack of awareness among faculty and staff further contribute to academic and psychosocial burden [3]. In addition to dietary restrictions, CeD is associated with substantial economic and social costs, including increased healthcare utilization, reduced productivity, and limitations in social participation [4].

CeD qualifies as a disability under the Americans with Disabilities Act (ADA). In 2008, Congress amended the ADA to clarify that covered major life activities include eating, as well as the operation of major bodily functions such as the immune and digestive systems. Because CeD directly impairs immune and digestive function and substantially limits the major life activity of eating, it meets the ADA’s definition of a disability [5]. Importantly, this legal classification does not suggest that individuals with CeD experience constant functional limitations; rather, it recognizes that, without reasonable accommodations, environmental barrier, particularly unsafe food access, can place students at risk of acute illness and long-term health consequences. When appropriate accommodations are in place, most students with CeD are able to fully participate in academic, residential, and social aspects of campus life.

Although legal protections exist under the ADA and Section 504 of the Rehabilitation Act, enforcement remains uneven across institutions. Recent legal actions, including the United States Department of Justice (DOJ) settlements with Lesley University and Rider University, as well as a public settlement of a lawsuit against the University of Maryland, highlight growing scrutiny and accountability for colleges failing to meet their obligations [6,7]. These cases underscore the urgent need for structured, evidence, and expert-informed recommendations to help institutions effectively support students with CeD.

Prior research examining CeD in the college setting has primarily focused on dietary adherence, quality of life, and perceived barriers to maintaining a GF diet [8,9,10]. Studies have documented challenges related to dining hall food safety, social isolation, hypervigilance around food choices, and reduced quality of life among students navigating campus environments [8]. However, there is a notable paucity of research addressing how colleges and universities operationalize accommodations for students with CeD across academic, housing, dining, and campus life domains. Existing studies rarely examine disability services processes, institutional policies, or cross-sector coordination between healthcare providers and higher education administrators. As a result, accommodations are often implemented inconsistently, leaving institutions without clear, expert-informed guidance and students without predictable support.

While the precise number of college students requesting nutrition-related accommodations (such as GF or allergen-safe diets) is not known, related data on disability and accommodation in higher education provides useful context. In the 2019–2020 academic year, approximately 21% of undergraduate students self-reported a disability, but only about 12–13% formally notified their institution and sought accommodations [11]. Among those who disclose, a high proportion (~85%) at 4-year colleges do receive accommodations [12]. These data suggest that the demand for accommodations may be broader than current utilization indicates. At the same time, scaling accommodations across diverse needs already places strain on institutional disability services [13]. The absence of published estimates specific to dietary accommodations underscores a critical knowledge gap and highlights the need for ongoing monitoring and reporting in the domain of medically required diet access in higher education.

Accordingly, the objective of this study was to develop the first ever expert-informed, consensus-based national recommendations to guide how colleges and universities ensure students with CeD have equitable access to education, safe food, housing, and campus experiences.

## 2. Materials and Methods

The Celiac Disease Foundation convened a national panel of multidisciplinary experts. Panelists were identified through a structured selection process designed to ensure both subject-matter expertise and broad representation of stakeholder perspectives.

Experts were nominated by the Society for the Study of Celiac Disease Education Committee based on demonstrated professional experience, publication record, or leadership in clinical care, research, education, disability law, or campus accessibility as it relates to students with CeD or other medically restricted diets. The expert panel also included higher-education stakeholders, including university disability services representatives, dining services leaders, and college students with CeD to capture on-the-ground implementation perspectives. The final group reflected geographic diversity across the United States and included representatives from both public and private institutions of varying sizes.

Of the 44 individuals invited, 40 accepted and agreed to participate in all phases of the consensus process. The panel included pediatric gastroenterologists (*n* = 11), clinical dietitians (*n* = 11), university/college-based foodservice providers (*n* = 5), a disability rights attorney (*n* = 1), higher education administrators (*n* = 1), patient advocates (*n* = 3), college students with CeD (*n* = 5), and parents of college students with CeD (*n* = 3).

Over a six-month period (10 February–4 August 2025), panel members engaged in a systematic literature review, stakeholder interviews, structured consensus-building exercises, and an in-person summit in Boston, Massachusetts.

A structured literature review was conducted prior to the panel meeting to identify existing evidence and guidance relevant to CeD management and accommodations in higher education settings. PubMed and Google Scholar were searched for English-language articles published between January 2000 and January 2025 using terms including CeD, GF diet, college, university, accommodation, disability services, and food service. This search yielded 11 relevant documents, including 8 peer-reviewed publications and 3 college guidance resources developed by nonprofit celiac disease support organizations. Key findings from this review were summarized and circulated to all panelists in advance of consensus discussions to ensure that deliberations were grounded in the best available evidence and expert experience.

To provide context and alignment with existing accommodation frameworks, the panel reviewed published guidelines and position statements related to food allergy and other medically indicated dietary restrictions in higher education and institutional food service settings. This included the College Food Allergy Guidelines developed by Food Allergy Research & Education (FARE) and associated implementation tools for campus professionals.

The Celiac Disease Foundation collaborated with FARE throughout the development process to promote consistency and complementarity between the two sets of recommendations. Representatives from both organizations participated in cross-review of draft materials, with a shared goal of developing co-endorsed guidance to support safe, inclusive, and practical food access for students with medically required diets.

An initial draft of CeD recommendations was developed collaboratively by the expert panel, followed by two rounds of structured online voting. Recommendations were revised based on panel feedback for clarity, feasibility, and consistency with clinical practice and legal standards. Given the paucity of interventional or comparative studies in this area, recommendations were based on expert consensus informed by the best available evidence from published literature, disability law, and institutional policy precedent. A recommendation was adopted only if it achieved at least 90% approval among panel members.

## 3. Results

The panel adopted 24 accommodations across four domains including academics, housing, dining, and campus life.

***Academic Accommodations***: Five recommendations were established to support students experiencing symptoms related to gluten exposure. These include academic flexibility through extended time on assignments or exams, modifications to food-related coursework, and remote access to class content. Additional accommodations included ensuring safe food environments during academic events and permitting students to leave class without penalty if symptomatic from a gluten exposure.

***Housing Accommodations***: Six recommendations were developed to reduce risk of gluten exposure in shared living environments and enhance accessibility. These include need-based priority housing placement, roommate considerations and agreements, proximity to safe dining locations, ability to bring select food appliances into dorm rooms, and access to low-use or private bathrooms.

***Dining Accommodations***: Seven recommendations address the need for consistent and safe GF meals. Dining accommodations include provision of reliable GF options, training for dining staff on CeD and cross-contact prevention, and full transparency in ingredient labeling. Additional accommodations include institutions offering direct communication channels with dining leadership, pre-ordered or custom meals, exemptions from mandatory meal plans when medically necessary, and access to dedicated GF food preparation areas.

***Campus Life Accommodations***: Six recommendations support broader participation in campus activities. These include ensuring GF food is available during athletic travel and events, providing support and flexibility for study abroad participation, offering safe food or permission to bring personal food at campus events, and supporting the mental health needs of students with CeD. An additional recommendation includes the integration of GF needs into emergency planning and a final accommodation to address internships or campus employment.

A complete summary of the 24 adopted accommodations is presented in Table 1.

Of the recommendations considered, all but one achieved at least 90% panel approval. The sole recommendation that did not reach consensus proposed that students with CeD be granted priority class registration to support meal planning and access to dining accommodations. While 78% of panelists endorsed the concept, others noted that priority registration policies differ across institutions and may be reserved for limited categories under disability services protocols. Panelists agreed that flexibility in class scheduling should be encouraged as part of individualized accommodation planning, but that a universal recommendation for priority registration could not be justified or feasibly implemented. Consequently, this item was excluded from the final recommendations.

## 4. Discussion

These suggested practices present the first set of national, expert-informed recommendations specifically focused on the needs of students with CeD in higher education. These recommendations address a critical gap in guidance for colleges and universities by providing a comprehensive framework for academic, housing, dining, and campus life accommodations. Accommodations should be individualized, as not every recommended modification is necessary or appropriate for every student. Although students with CeD are legally entitled to disability accommodations under the ADA and Section 504 of the Rehabilitation Act, implementation across institutions is inconsistent, and students frequently report inadequate support [14]. This inconsistency, combined with the substantial burden of maintaining strict adherence to a GF diet and the associated hypervigilance required to avoid exposure, has been shown to negatively affect quality of life among adolescents with CeD and may carry forward into the higher education environment [8].

While CeD qualifies as a disability under the ADA, it is important to distinguish legal classification from lived experience. Many students with CeD experience no day-to-day functional limitations when they are able to strictly adhere to a GF diet. The role of accommodations in this context is therefore not to remediate ongoing impairment, but to prevent avoidable gluten exposure that can trigger acute illness, disrupt academic participation, and compromise long-term health. In this way, accommodations operate as a mechanism to prevent discrimination by removing barriers that would otherwise exclude students with CeD from equal participation in activities that could lead to a gluten exposure.

It is important to recognize that accommodations for students with CeD are not simply dietary preferences, but essential supports that address functional limitations associated with an autoimmune condition. Ingestion of gluten, even in trace amounts, can cause acute gastrointestinal symptoms such as abdominal pain, diarrhea, vomiting, and nausea, as well as fatigue and cognitive impairment [1]. These symptoms may arise suddenly following inadvertent exposure and can significantly interfere with class attendance and participation. Providing access to private or nearby bathroom facilities helps preserve dignity and reduces the risk of embarrassment or academic disruption. Similarly to accommodations routinely provided for students with other chronic medical conditions (e.g., inflammatory bowel disease, diabetes), these measures ensure equitable participation in educational activities and campus life. Such accommodations align with the intent of the ADA and Section 504 of the Rehabilitation Act, which require that institutions mitigate barriers related to chronic health conditions through reasonable, individualized supports [5].

Operational models differ widely across United States campuses, with many universities outsourcing dining operations to corporate providers. The obligation to accommodate applies regardless of how dining services are structured. Even when universities contract with third-party vendors, the institution remains responsible for ensuring that students with CeD receive reasonable accommodations and have access to safe GF options consistent with ADA and Section 504 requirements.

Study abroad participation was recognized as an important dimension of equitable access, though it is influenced by financial, programmatic, and geographic constraints. The panel does not expect international programs to provide identical accommodation infrastructure. Rather, institutions should take reasonable steps to help students with CeD or other conditions requiring a medically necessary GF diet assess feasibility and understand potential risks. This may include evaluating GF food availability, communicating limitations, and offering alternative or comparable academic options when adequate support cannot be ensured. This approach aligns with ADA principles by balancing inclusion with practical feasibility and enabling students to make informed decisions.

We acknowledge that a substantial number of students follow GF diets without confirmed CeD, which may increase institutional demand for GF options. While these recommendations focus primarily on students with CeD, for whom a strict GF diet is medically necessary, they may also be relevant to students with other medical conditions requiring GF diets (e.g., non-celiac gluten sensitivity, eosinophilic esophagitis), which can typically be accommodated with documentation from a treating clinician. Institutions generally require a physician’s letter verifying medical necessity to support such requests.

The development of these recommendations was guided by a diverse expert panel, whose perspectives spanned clinical care, legal rights, dietary management, and lived experience. The consensus summit presentations informed key components of the recommendations and highlighted the urgency of institutional change. Mental health professionals and dietitians emphasized the psychosocial toll of living with CeD on campus, including fear of cross-contact, food insecurity, and social isolation, all of which can erode quality of life and educational engagement.

The recommendations were also shaped by legal insights demonstrating that most students with CeD do not have formal accommodation plans, despite meeting criteria for coverage. Table 2 presents a summary of the legal obligations and relevant case precedents supporting institutional accommodations for students with CeD. Additionally, personal stories from students participating in the consensus summit exposed the real-world failures of current systems and underscored the need for policies that are practical, enforceable, and student-centered. Equally important, input from college food service personnel grounded the recommendations in operational feasibility. Their expertise confirmed that the proposed accommodations are not only reasonable but are already being successfully implemented on a limited number of campuses. These perspectives helped ensure that the final recommendations are medically and legally sound, while also achievable in day-to-day practice.

Although these recommendations aim to guide proactive and effective institutional support, students may still encounter challenges in securing appropriate accommodations. If students encounter difficulty obtaining or maintaining reasonable accommodations, a tiered escalation approach is recommended. First, students should collaborate with disability services to revise their existing plan. When additional clinical justification is needed, documentation provided by a treating physician or registered dietitian can support accommodation requests. If barriers persist, students may seek assistance from patient advocacy organizations or, as a last resort, consult with a disability rights attorney specializing in postsecondary access. Together, these pathways help ensure students receive timely support and guidance.

## 5. Conclusions

By codifying expert-informed best practices, these recommendations serve as a critical resource for healthcare providers, disability services personnel, dining services, and administrators. Adoption of these recommendations offers a path toward improving health outcomes and fostering more equitable and inclusive learning environments for students with CeD. Future directions should include pilot implementation at multiple campuses, collection of outcomes data on student success and well-being, and development of tools to track compliance and impact.

## Figures and Tables

**Table 1:** Summary of 24 Recommended Accommodations for Students with Celiac Disease (CeD) in Higher Education.

Domain	Code	Accommodation	Description
**Academic**	A1	Extended Time	Additional time on assignments or exams during recovery from gluten exposure.
	A2	Modified Coursework	Substitutions or protections in food-related labs or courses to prevent gluten exposure.
	A3	Gluten-Free (GF) Class Environments	Safe GF options when food is served or incorporated into academic activities.
	A4	Leave Without Penalty	Permission to leave class if symptomatic from gluten exposure without academic penalty.
	A5	Remote Access	If a student is unable to attend class due to illness from gluten exposure, access to recorded lectures, asynchronous materials, or remote participation options should be made available when possible.
**Housing**	H1	Priority Housing	Considered for early or prioritized selection for housing that supports safe GF living.
	H2	Roommate Considerations	Ability to request or match with roommates who understand and support dietary needs.
	H3	Dining Proximity	Housing located near safe GF dining options.
	H4	Bathroom Access	Access to a nearby single or low-use bathroom is preferred. If communal bathrooms are used, students should be placed in rooms that have bathrooms conveniently located and easily accessible nearby.
	H5	Kitchen Appliances	Reliable access to safe food storage and preparation appliances in accordance with university housing and electrical safety regulations.
	H6	Transportation Flexibility	Car access exemptions for food shopping and medical appointments.
**Dining**	D1	Reliable GF Meals	GF meals available during all dining hours, including weekends and holidays.
	D2	Staff Training	Dining staff trained on CeD, GF preparation, and cross-contact prevention.
	D3	Ingredient Transparency	Clear labeling and access to full ingredient lists and allergen information.
	D4	Direct Communication	Students have access to dietitians, dining managers, and lead chef for support and issue resolution.
	D5	Custom Meals	Option for pre-ordered or customized meals prepared in cross-contact–free environments.
	D6	Meal Plan Exemption	Option to opt out of meal plans when safe food cannot be reliably provided.
	D7	Dedicated GF Zones	Use of GF prep areas, equipment, and storage when possible.
**Campus Life**	C1	Athletic Support	Access to GF food and flexibility during team events or after illness.
	C2	Study Abroad Inclusion	Support for safe food access, housing with a kitchen, and flexibility in placement.
	C3	Social Event Access	GF options or permission to bring personal food to campus events. Education for event leaders.
	C4	Mental Health Support	Recognition of the psychosocial impact with referrals to campus counseling.
	C5	Emergency Planning	Protocols to ensure GF food and medical access during emergencies or shelter in place.
	C6	Internship and Employment Flexibility	Accommodations for GF food and illness management during campus employment or internships.

**Table 2:** Legal Obligations and Precedent Supporting Celiac Disease (CeD) Accommodations in Higher Education.

Case Examples	Key Provisions	Implications for Institutions	Legal Framework
Department of Justice (DOJ) Settlement: Rider University (2019)—failure to accommodate a gluten-free (GF) diet violated civil rights.	Requires accommodations for individuals with disabilities, including those with CeD.	Institutions should recognize CeD as a disability and provide individualized accommodations.	Americans with Disabilities Act (ADA)
DOJ Settlement: Lesley University (2013)—required comprehensive GF dining plan reform	Prohibits discrimination based on disability in federally funded programs.	Dining services should offer accessible, medically safe meals and an interactive accommodation process.	ADA
Public Settlement: Smith v. University of Maryland (2020)—student filed suit after repeated gluten exposures in campus dining led to medical harm and academic disruption. Resulted in $200,000 settlement.	Clarifies requirements for non-discrimination and equal access for students with disabilities.	Institutions should establish proactive systems to document, evaluate, and respond to medically necessary dietary accommodations. As a result of the settlement, the University of Maryland conducted a third-party dining audit to ensure safe access to GF meals for students.	Section 504 of the Rehabilitation Act ADA

## Data Availability

The original contributions presented in this study are included in the article and Supplementary Materials. Further inquiries can be directed to the corresponding author.

## Supplementary Materials

The complete National Recommendations for Supporting Students with Celiac Disease in Higher Education will be available at www.celiac.org/school (accessed on 1 January 2026) upon publication of this manuscript. The website also currently hosts the 2019 Voluntary Recommendations for Managing Celiac Disease in Learning Environments (K–12).

## Abbreviations

The following abbreviations are used in this manuscript:

CeD	Celiac Disease
GF	Gluten-Free
FARE	Food Allergy Research & Education
ADA	Americans with Disabilities Act
DOJ	Department of Justice
